# Spatial Autocorrelation Approaches to Testing Residuals from Least Squares Regression

**DOI:** 10.1371/journal.pone.0146865

**Published:** 2016-01-22

**Authors:** Yanguang Chen

**Affiliations:** Department of Geography, College of Urban and Environmental Sciences, Peking University, 100871, Beijing, China; University California Los Angeles, UNITED STATES

## Abstract

In geo-statistics, the Durbin-Watson test is frequently employed to detect the presence of residual serial correlation from least squares regression analyses. However, the Durbin-Watson statistic is only suitable for ordered time or spatial series. If the variables comprise cross-sectional data coming from spatial random sampling, the test will be ineffectual because the value of Durbin-Watson’s statistic depends on the sequence of data points. This paper develops two new statistics for testing serial correlation of residuals from least squares regression based on spatial samples. By analogy with the new form of Moran’s index, an autocorrelation coefficient is defined with a standardized residual vector and a normalized spatial weight matrix. Then by analogy with the Durbin-Watson statistic, two types of new serial correlation indices are constructed. As a case study, the two newly presented statistics are applied to a spatial sample of 29 China’s regions. These results show that the new spatial autocorrelation models can be used to test the serial correlation of residuals from regression analysis. In practice, the new statistics can make up for the deficiencies of the Durbin-Watson test.

## Introduction

Least squares regression can be employed to make models of real systems and to reveal the hidden relationships between causes and effects. The major aims of mathematical modeling lie in explanation and prediction, which are sometimes contradictory [1–3]. By means of regression modeling, we can explain the causes of an effects or predict the effects with causes. The quality of a mathematical model depends on its structure. A model must simplify reality to the moment. As Longley [4] pointed out: “In the most general terms, a ‘model’ can be defined as a ‘simplification of reality’, nothing more, nothing less.” Both oversimplification (e.g., explanatory variables are incomplete) and undersimplification (e.g., explanatory variables are redundant) of reality can lead to trustless explanation and unfaithful prediction. The structural problems of a model can be reflected by residuals, that is, a series of errors between observed values from the real world and predicted values given by the model. A good model will yield a random series of residuals without autocorrelation. The residual series free of autocorrelation satisfies the normal distribution. Autocorrelation in the residual series suggests an inherent defect in the model.

The theory of time series analysis can be employed to detect serial correlation of residuals from linear regression [5]. The time series analysis can be generalized to ordered space series analysis [6, 7]. However, many scientists do not have enough knowledge of time/space series analysis. Thus it is necessary to invent a simple statistic for testing serial correlation in least squares regression. Maybe the simplest approach to residual autocorrelation analysis is the Durbin-Watson test. Durbin and Watson [8–10] wrote a series of articles on a method of testing for serial correlation in a regression analysis. One of the fruits is the well-known Durbin-Watson’s statistic, which is easy to understand, calculate, and explain. It is in fact a simple index, by which residual serial correlation information can be concentrated into a concise number. Years later, the serial correlation test was advanced by modifying the standard Durbin-Watson assumptions [11]. However, Durbin-Watson’s method and its derivatives have a significant limitation: that is, they cannot be applied to regression analysis based on cross-sectional data, which is defined in a two-dimensional space. The Durbin-Watson formula is constructed with one-order time lag or one-step space displacement. Therefore, it is only applicable to least squares regressions based on ordered time series or spatial series, which are defined in a one-dimensional space. An ordered time series or spatial series has exclusive rank for observed data of a variable, and thus the result of Durbin-Watson statistic is uniquely determined. However, an array of cross-sectional data can have various arrangement orders. Changing the rank of elements in an array will result in a different series of residuals and thus lead to many different values of the Durbin-Watson statistic. In particular, in geographical analysis, lots of least squares regressions are based on cross-sectional data from spatial random sampling. The Durbin-Watson test is often ineffective in the linear regression of spatial variables.

An effective way of testing the serial correlation of residuals from least squares regression based on cross-sectional data is to make use of spatial autocorrelation analysis. Actually, the core of the formula of Durbin-Watson’s statistic is just a one-dimensional autocorrelation coefficient. Using a weight function to replace the time-lag parameter or space-displacement parameter, the one-dimension spatial or temporal autocorrelation model can be generalized to a two-dimensional spatial autocorrelation model [12]. There are two basic and important statistics for spatial autocorrelation analysis: Moran’s index [13] calculated by generalizing Pearson’s correlation coefficient, and Geary’s coefficient [14], constructed by analogy with Durbin-Watson’s statistic. Using residuals and the corresponding spatial contiguity, we can calculate Moran’s index and thus judge the serial correlation for a regression model [15, 16]. The Moran’s index of spatial residuals is analogous to the autocorrelation coefficient of a time series of errors. However, the autocorrelation coefficient is not convenient for testing serial correlation of temporal residuals, and thus we need Durbin-Watson statistic [8]. In like manner, Moran’s index is not easy for testing serial correlation of random spatial residual series, and thus we need new spatial statistic measurements. The simple statistics of testing for spatial serial correlation can be defined by means of Moran’s index or by analogy with Geary’s coefficient.

The aim of this study is to develop simple methods to test residuals of regression analysis based on spatial data from a new point of view. Moran’s index proved to be expressed in a compact equation using a standardized vector and a normalized spatial weight matrix [12, 17]. Based on the new mathematical expression of Moran’s index, a relatively precise formula of the residual autocorrelation test can be defined. Further, by analogy with Geary’s coefficient, an approximate expression of the residual autocorrelation measurement can be put forward. The rest of this paper is organized as follows: In Section 2, the basic expressions of the two-dimensional spatial autocorrelation of residuals are given for measuring serial correlation, and a residual autocorrelation scatterplot is proposed for making supporting analyses; In Section 3, a set of case studies shows how to utilize the methods presented in this work to test for serial correlation; In Section 4, the two-dimensional spatial autocorrelation measurement of residuals is generalized and developed, and the deficiency of these measurements is discussed. Finally, the paper concludes with summarizing the highlights of this study.

## Models and Methods

### 1. A deficiency in the Durbin-Watson test

It is necessary to explain the ordinary linear regression model and its predicted residuals. Suppose there are *m* variables (*j* = 1, 2, …, *m*) and *n* spatial elements in a region (*i* = 1, 2, …, *n*). In this instance, the sample size is *n*. The multivariable linear regression equation can be expressed as

$$y_{i}=a+\sum_{j=1}^{m}b_{j}x_{ij}+\varepsilon_{i},$$ (1)

where *x*_*i*_ denotes independent variables (input variables, explanatory variables, arguments), *y*_*i*_ represents a dependent variable (output variable, explained variable, function), *a* refers to a constant (intercept), *b*_*j*_ to regression coefficients (slopes), and *ε*_*i*_ to residuals (predicted errors). The residuals are supposed to be a white noise series and must satisfy the following conditions

$$\varepsilon_{i}\sim \text{WN}(0,\sigma^{2}),$$ (2)

where “WN” means “white noise”, and *σ* denotes the standard deviation of the residual series. That is to say, the average value of the residual series must be 0, and its limited variance is a constant *σ*^2^. If and only if the residual series is white noise, it will imply that the errors between the observed values and the corresponding predicted values of the regression model, Eq 1, come from random disturbances outside the model. Otherwise, it will mean that the errors result from the internal structure of the model itself. One approach to judging whether or not the residual series is white noise is the well-known Durbin-Watson statistic (ab. DW) [8–10], which is defined as

$$DW=\frac{\sum_{i=2}^{n}{\left(\varepsilon_{i}-\varepsilon_{i-1}\right)}^{2}}{\sum_{i=1}^{n}\varepsilon_{i}^{2}}=\frac{\sum_{i=1}^{n-1}{\left(\Delta \varepsilon_{i}\right)}^{2}}{\sum_{i=1}^{n}\varepsilon_{i}^{2}}=2(1-\rho ),$$ (3)

where Δ*ε*_*i*_ = *ε*_*i*_-*ε*_*i-1*_, and

$$\rho =\frac{\sum_{i=2}^{n}\varepsilon_{i}\varepsilon_{i-1}}{\sum_{i=1}^{n}\varepsilon_{i}^{2}}$$ (4)

denotes the autocorrelation coefficient of the residual series, which must be a time series or an ordered space series. In Eqs 3 and 4, the difference of *i* indicates a one-order time lag (*k* = Δ*i* = 1) or a one-step space displacement (*r* = Δ*i* = 1). Because the autocorrelation coefficient *ρ* comes between -1 and 1 (i.e., -1≤*ρ*≤1), the *DW* values vary from 0 to 4 (i.e., 0≤*DW*≤4). If the residuals have no serial correlation, then *ρ* = 0, and thus *DW* = 2. This suggests that if the Durbin-Watson statistic is close to 2, the residual series can be regarded as free of autocorrelation at a certain significance level (say, *α* = 0.05).

However, the Durbin-Watson test is only applicable to the serial correlation of residuals from the least squares regression based on times series, for example, the US level of urbanization from 1790 to 2010, or ordered spatial series like the average urban population density of the rings from the center of a city to its exurbs [18]. If we perform a regression analysis using cross-sectional data coming from spatial random samples, the residuals will form a random space series, and thus the Durbin-Watson method will be ineffective. As indicated by Eq 3, the *DW* value is calculated with residuals and the sum of squares of the differences of residuals, but the sum of squares of the residual differences depends on the arrangement of elements in a random sample. For cross-sectional data, the elements can be arranged in a spreadsheet at random. The results from different data arrangements will differ from one another. For example, suppose that for the set of elements (A, B, C), the corresponding array is (1, 2, 3). Thus the vector of difference is (1, 1), and the sum of squares of the differences is 2. If this is an ordered temporal or spatial set, the order of A, B, and C cannot be changed. However, for a spatial random sampling, the arrangement of the elements is arbitrary. If the elements are permuted and the result is (B, A, C), the corresponding array will change to (2, 1, 3). Then the difference vector is (-1, 2), and the sum of squares of the differences is 5. This suggests that, for a spatial random sample, the *DW* value is not certain. It depends on the arrangement of the elements in a set. In short, the current Durbin-Watson test can be applied to least squares regression based on time series or ordered space series but cannot be effectively used to test the residual serial correlation for regression analyses based on random space series.

### 2. An approach to test random serial correlation

An effective approach to solving this problem is to make proper use of spatial autocorrelation. Moran’s index is in fact a spatial autocorrelation coefficient. The mathematical expression of Moran’s index has been simplified by means of standardized vectors and a unitized matrix [12]. Using the normalized form of the formula for Moran’s index, we can construct new statistics for testing serial correlation of the least squares regression residuals based on spatial random samples. The series of residuals from prediction of Eq 1 can be standardized with the formula

$$e_{i}=\frac{\varepsilon_{i}}{\sigma },$$ (5)

where *σ* refers to the standard deviation of the predicted residuals. If the spatial distance matrix of the random sampling points has been obtained, we will have an *n*-by-*n* unitary spatial weights matrix (SWM) such as

$$W={\left[w_{ij}\right]}_{n\times n}.$$ (6)

The three properties of this matrix are as follows: (1) Symmetry, i.e., *w*_*ij*_ = *w*_*ji*_; (2) Zero diagonal elements, i.e., *w*_*ii*_ = 0, meaning that the entries in the diagonal are all 0; and (3) Unitary condition, that is

$$\sum_{i=1}^{n}\sum_{j=1}^{n}w_{ij}=1.$$ (7)

Thus the spatial autocorrelation coefficient of the residuals can be computed by the following formula

$$I=e^{\text{T}}We,$$ (8)

where *I* denotes spatial autocorrelation index (SAI) of residuals. The SAI is equivalent to Moran’s index of spatial residuals. The index ranges from -1 to 1 (i.e., -1≤*I*≤1). If the residuals have no serial correlation, we will have *I* = 0. By analogy with the Durbin-Watson statistic expressed with Eq 3, the residual correlation index (RCI) of the least squares regression can be defined as

S=2(1−I), (9)

where *S* indicates RCI. The *S* value comes between 0 and 4 (i.e., 0≤*S*≤4). If *I* = 0, then *S* = 2. So, if the *S* value is close to 2, we will reach a conclusion that the residuals have no spatial autocorrelation according to a certain significance level.

A residual autocorrelation scatterplot can be constructed for serial correlation analysis by analogy with the normalized Moran’s scatterplot. Owing to *e*^T^*e* = *n*, Eq 8 can be expressed as

$$e^{\text{T}}eI=e^{\text{T}}(nW)e,$$ (10)

This suggests the precondition that Eq 10 comes into existence is as follows

nWe=Ie. (11)

Apparently, Eq 8 can be derived from Eq 11. On the other hand, Eq 8 multiplied left by *e* on both sides of the equal sign yields

$$ee^{\text{T}}We=Ie.$$ (12)

Based on Eq 11, a random variable can be defined for observed values in the form

$$y=e^{\text{T}}eWe=nWe.$$ (13)

Based on Eq 12, a trend variable can be defined for predicted values as below

$$\hat{y}=ee^{\text{T}}We=Ie.$$ (14)

Then, using *e* as *x*-axis with *y* and *ŷ* as *y*-axis, we can make a serial correlation scatterplot. In the plot, the relationship between *e* and *y* gives the scattered points, and the relationship between *e* and *ŷ* yields the trend line. The slope of the trend line is equal to the SAI value.

### 3. Developed and alternative mathematical forms

A mathematical model or a statistical measurement has two expressions: one is based on the population (universe), and the other is based on samples. The former applies to mathematical transformation or theoretical reasoning, and the latter applies to empirical analyses. For example, we have population standard deviation (PSD) and sample standard deviation (SSD). Thus we have two sets of principal component analysis (PCA): one is based on the PSD, and the other is based on the SSD [5]. If we are trying to develop PCA theory, we can use the former, and if we are attempting to do positive research, we should use the latter. Both the theoretical expression and the empirical expression of a mathematical model can be applied to empirical studies. If a sample is large enough, the results and conclusions will be the same. However, if a sample is small, the empirical expression of a mathematical model will be superior to the corresponding theoretical expression. In spatial autocorrelation theory, Moran’s index is based on the PSD, while Geary’s coefficient is based on the SSD. In other words, to compute Moran’s index, the variable standardization is based on the PSD; however, to calculate Geary’s coefficient, the variable standardization is based on the SSD [12]. Of course, it is easy to construct Moran’s index by means of SSD.

A theoretical definition of RCI and the related analytical process for random spatial serial correlation have been proposed above. In fact, the RCI can be defined in two forms: one is the relatively precise form based on Moran’s index, the other is the approximate form based on Geary’s coefficient. In theory, the RCI is expressed in the form based on the population; while in practice, it always takes the form based on a sample. No matter what form it is, a spatial contiguity matrix (SCM) must be constructed [19]. Suppose there are *n* elements in a geographic region. A SCM can be expressed as

$$V={\left[v_{ij}\right]}_{n\times n}=\left[\begin{array}{cccc} v_{11} & v_{12} & \cdots & v_{1n}\\ v_{21} & v_{22} & \cdots & v_{2n}\\ \vdots & \vdots & \ddots & \vdots \\ v_{n1} & v_{n2} & \cdots & v_{nn} \end{array}\right],$$ (15)

where *V* denotes the SCM, *v*_*ij*_ is a measure used to compare and judge the degree of nearness or the contiguous relationships between location *i* and location *j* (*i*, *j* = 1, 2, …, *n*). The elements on the diagonal are zeros (i.e., for *i* = *j*, *v*_*ii*_≡0). A sum of SCM entries can be defined as

$$T=\sum_{i=1}^{n}\sum_{j=1}^{n}v_{ij}.$$ (16)

The SCM can be converted into SWM by the following formula:

$$w_{ij}=\frac{v_{ij}}{T}=v_{ij}/\sum_{i=1}^{n}\sum_{j=1}^{n}v_{ij},$$ (17)

by which the SCM can be made unitary by matrix (completely unitary). In literature, the SCM is always made unitary by row (locally unitary). This is not desirable because the result will violate the distance axiom [5]. A SWM is actually based on a generalized spatial distance matrix, which must satisfy the axiom of distance; otherwise it is unacceptable.

Using the ideas from spatial autocorrelation, we can derive a set of new indices for testing serial correlation in the least squares regression of spatial random samples. Based on population, Eq 8 can be developed in detail to yield an expression similar to the formula of Moran’s index, that is

$$I_{\text{P}}=\frac{\varepsilon^{\text{T}}(nW)\varepsilon }{\varepsilon^{\text{T}}\varepsilon }=\frac{n\sum_{i=1}^{n}\sum_{j=1}^{n}v_{ij}(\varepsilon_{i}-\mu )(\varepsilon_{j}-\mu )}{T\sum_{i=1}^{n}{\left(\varepsilon_{i}-\mu \right)}^{2}}=\frac{n\sum_{i=1}^{n}\sum_{j=1}^{n}w_{ij}\varepsilon_{i}\varepsilon_{j}}{\sum_{i=1}^{n}\varepsilon_{i}^{2}}.$$ (18)

where *μ* = 0 denotes the mean of residuals. Eq 18 is based on PSD. Thus the RCI based on population can be expressed as

$$S_{\text{p}}=2(1-I_{\text{p}}),$$ (19)

which is suitable for theoretical analyses rather than empirical studies. Based on samples, Eq 18 can be revised as

$$I_{\text{s}}=\frac{\varepsilon^{\text{T}}[(n-1)W)]\varepsilon }{\varepsilon^{\text{T}}\varepsilon }=\frac{(n-1)\sum_{i=1}^{n}\sum_{j=1}^{n}w_{ij}\varepsilon_{i}\varepsilon_{j}}{\sum_{i=1}^{n}\varepsilon_{i}^{2}},$$ (20)

which indicates a new Moran’s index, which is based on SSD. Accordingly, the RCI based on samples can be expressed as

$$S_{\text{s}}=2(1-I_{\text{s}}).$$ (21)

This formula can be applied to the empirical analyses of least squares regression based on the smaller spatial samples.

As indicated above, Geary’s coefficient is similar to the Durbin-Watson statistic in mathematical principle. Now, by analogy with the formula of Geary’s coefficient, we can define serial autocorrelation index in the following form

$$C=\frac{(n-1)\sum_{i=1}^{n}\sum_{j=1}^{n}v_{ij}{\left(\varepsilon_{i}-\varepsilon_{j}\right)}^{2}}{2\sum_{i=1}^{n}\sum_{j=1}^{n}v_{ij}\sum_{i=1}^{n}{\left(\varepsilon_{i}-\mu \right)}^{2}}=\frac{(n-1)\sum_{i=1}^{n}\sum_{j=1}^{n}w_{ij}{\left(\varepsilon_{i}-\varepsilon_{j}\right)}^{2}}{2\sum_{i=1}^{n}\varepsilon_{i}^{2}}.$$ (22)

Thus an approximate residual correlation index (ARCI) can be defined as

$$S_{a}=2C=\frac{(n-1)\sum_{i=1}^{n}\sum_{j=1}^{n}w_{ij}{\left(\varepsilon_{i}-\varepsilon_{j}\right)}^{2}}{\sum_{i=1}^{n}\varepsilon_{i}^{2}},$$ (23)

which is based on SSD. This formula is suitable for the positive studies by means of the least squares regression based on the larger spatial samples.

Both the RCI and ARCI can be termed *spatial Durban-Watson* (SDW) statistics. In theory, we have

$$S_{a}=2(1-I_{s})=S_{s}.$$ (24)

However, in practice, we have

$$S_{a}\approx 2(1-I_{s})=S_{s}.$$ (25)

This can be demonstrated by means of mathematical transformation. Please note that a mathematical proof is always based on PSD rather than SSD. The derivation is as follows:

$$\begin{array}{cc} S & =2C=\frac{n\sum_{i=1}^{n}\sum_{j=1}^{n}w_{ij}{\left(\varepsilon_{i}-\varepsilon_{j}\right)}^{2}}{\sum_{i=1}^{n}\varepsilon_{i}^{2}}=\frac{2n\sum_{i=1}^{n}\sum_{j=1}^{n}w_{ij}(\varepsilon_{i}^{2}-\varepsilon_{i}\varepsilon_{j})}{\sum_{i=1}^{n}\varepsilon_{i}^{2}}\\ & =2[\frac{\sum_{i=1}^{n}\sum_{j=1}^{n}w_{ij}\varepsilon_{i}^{2}}{\frac{1}{n}\sum_{i=1}^{n}\varepsilon_{i}^{2}}-\frac{n\sum_{i=1}^{n}\sum_{j=1}^{n}w_{ij}\varepsilon_{i}\varepsilon_{j}}{\sum_{i=1}^{n}\varepsilon_{i}^{2}}]\\ & \approx 2[1-I_{\text{p}}]. \end{array}$$ (26)

Here the arithmetic mean value of the squared errors is close to the weighted average of the squared residuals for a population or a large sample. If the population is replaced by a sample, the measurement *I*_p_ will be substituted with the index *I*_s_.

In fact, the Durbin-Watson statistic is an approximate measure rather than an exact measure for serial correlation. ARCI is more similar to the DW index than RCI. Comparing Eq 23 with Eq 3 shows that there is a clear analogy between the Durbin-Watson statistic and the ARCI. The difference rests with that the one-order time lag in Eq 3 is replaced by a spatial weight function in Eq 23. For an even distribution of *n* elements and if *n* is very large, we will have a weight *w*_*ij*_→1/*n*≈1/(*n*-1). The spatial difference *ε*_*i*_ −*ε*_*j*_ is analogous to the temporal difference *ε*_*i*_-*ε*_*i-1*_. This suggests the Durbin-Watson formula defined by Eq 3 and the ARCI defined by Eq 23 are mathematically isomorphic to each other.

## Case Study

### 1. Study area, problems, and the analytical process

The method of spatial serial autocorrelation analysis can be applied to the least squares regression of the relationship between urbanization and economic development. This relationship can be modeled with a nonlinear function such as logarithmic function [20], but an approximate analysis can be made using a linear equation. The study area is the mainland of China, which includes 31 provinces, autonomous regions, and municipalities directly under the Central Government of China. Two variables are employed to make the regression analysis: one is the *level of urbanization*, and the other, per capita *gross regional product* (GRP). The level of urbanization refers to the proportion of urban population to total population in a region. The statistical data of urbanization levels and per capita GRP (2000–2013) are available from the website of National Bureau of Statistics (NBS) of the People's Republic of China (http://www.stats.gov.cn/tjsj/ndsj/). In order to implement the spatial serial correlation test, we need a spatial contiguity matrix. The matrix can be generated with the distances by train between any two capital cities of regions. The railroad distance matrix can be found in many traffic atlases of China. Because the cities of Haikou and Lhasa are not connected to the network of Chinese cities by railway from 2000 to 2013, only 29 regions and their capital cities are taken into consideration, and thus the size of each spatial sample is *n* = 29 (Table 1). The datasets of urbanization level, per capita GRP, and railway distance are attached (datasets in S1 File). For the sample analysis, the number, *n*, should be replaced by the total degree of freedom, *n*-1 = 28 [12]. Since the number of independent variable is *m* = 1, the residual degree of freedom is *df* = *n*-*m*-1 = 27.

**Table 1:** The datasets of per capita GRP, level of urbanization, and the standardized residuals from linear squares regression of 29 Chinese regions (2012).

Arrangement in conventional order				Arrangement in alphabetical order			
Region	per capita GRP	Level of urbanization	Residual	Region	per capita GRP	Level of urbanization	Residual
**Beijing**	87475	86.20	0.9550	**Anhui**	28792	46.50	0.4496
**Tianjin**	93173	81.55	-0.9400	**Beijing**	87475	86.20	0.9550
**Hebei**	36584	46.80	-0.6196	**Chongqing**	38914	56.98	1.3671
**Shanxi**	33628	51.26	0.8315	**Fujian**	52763	59.60	-0.0564
**Inner Mongolia**	63886	57.74	-2.1058	**Gansu**	21978	38.75	-0.3268
**Liaoning**	56649	65.65	0.7591	**Guangdong**	54095	67.40	1.5320
**Jilin**	43415	53.70	-0.0399	**Guangxi**	27952	43.53	-0.1066
**Heilongjiang**	35711	56.90	1.8165	**Guizhou**	19710	36.41	-0.5305
**Shanghai**	85373	89.30	1.9705	**Hebei**	36584	46.80	-0.6196
**Jiangsu**	68347	63.00	-1.5549	**Heilongjiang**	35711	56.90	1.8165
**Zhejiang**	63374	63.20	-0.7830	**Henan**	31499	42.43	-0.8760
**Anhui**	28792	46.50	0.4496	**Hubei**	38572	53.50	0.6216
**Fujian**	52763	59.60	-0.0564	**Hunan**	33480	46.65	-0.2006
**Jiangxi**	28800	47.51	0.6793	**Inner Mongolia**	63886	57.74	-2.1058
**Shandong**	51768	52.43	-1.5500	**Jiangsu**	68347	63.00	-1.5549
**Henan**	31499	42.43	-0.8760	**Jiangxi**	28800	47.51	0.6793
**Hubei**	38572	53.50	0.6216	**Jilin**	43415	53.70	-0.0399
**Hunan**	33480	46.65	-0.2006	**Liaoning**	56649	65.65	0.7591
**Guangdong**	54095	67.40	1.5320	**Ningxia**	36394	50.67	0.2927
**Guangxi**	27952	43.53	-0.1066	**Qinghai**	33181	47.44	0.0236
**Chongqing**	38914	56.98	1.3671	**Shaanxi**	38564	50.02	-0.1726
**Sichuan**	29608	43.53	-0.3484	**Shandong**	51768	52.43	-1.5500
**Guizhou**	19710	36.41	-0.5305	**Shanghai**	85373	89.30	1.9705
**Yunnan**	22195	39.31	-0.2305	**Shanxi**	33628	51.26	0.8315
**Shaanxi**	38564	50.02	-0.1726	**Sichuan**	29608	43.53	-0.3484
**Gansu**	21978	38.75	-0.3268	**Tianjin**	93173	81.55	-0.9400
**Qinghai**	33181	47.44	0.0236	**Xinjiang**	33796	43.98	-0.8571
**Ningxia**	36394	50.67	0.2927	**Yunnan**	22195	39.31	-0.2305
**Xinjiang**	33796	43.98	-0.8571	**Zhejiang**	63374	63.20	-0.7830
**DW statistic**	2.2463			**DW statistic**	1.9071		
**RCI**	2.1830			**RCI**	2.1830		
**ARCI**	2.1435			**ARCI**	2.1435		

**Note**: The unit of the level of urbanization is percent (%), and the unit of GRP is *yuan* of Renminbi (RMB).

The analytical process consists of two operations: the first is the regression modeling of the levels of urbanization and economic development, which yields a series of predicted residuals; the second is the serial correlation test of residuals, which is based on spatial autocorrelation analysis. Using per capita GRP as an independent variable and the level of urbanization as a dependent variable, we can make a regression analysis easily. The regression results include residuals (*ε*_*i*_) as well as standardized residuals (*e*_*i*_). Then, the three-step calculation method, which is designed for computing Moran’s index [12], can be utilized to calculate the SAI. In order to figure out RCI with SAI, the three-step calculation method should be replaced by the four-step calculation method. The process comprising four steps is as follows. **Step 1: Standardize the residual vector**. The residual vector *ε* has been turned into the standardized vector *e* using Eq 5. In fact, the standardized residuals can be directly provided by MS Excel and SPSS. **Step 2: calculate the normalized SWM**. The railway distance matrix can be turned into a SCM with a weight function such as *v*_*ij*_ = 1/*r*_*ij*_, where *r*_*ij*_ refers to the railway distance between city *i* and city *j*, and *v*_*ij*_ to spatial contiguity of the two cities [19]. Then by means of Eqs 16 and 17, the SCM can be transformed into a unitary SWM, *W*. **Step 3: compute SAI**. In terms of Eq 8, the SWM *W* is first left multiplied by the transposition of *e*, and then the product of *e*^T^ and *W* is right multiplied by *e*. The final product of the continued multiplication is the SAI value. **Step 4: work out RCI**. It is very easy to calculate the RCI value by using Eq 9.

### 2. Testing for serial correlation of linear regression analyses

The correlation between the level of urbanization and the level of economic development is currently a hot topic in China. Linear regression analysis can be employed to study the relationship between urbanization and economic development. Using the per capita GRP indicative of economic development level as an argument (*G*) and the proportion of urban population indicative of the level of urbanization as a response variable (*L*), we can build a simple linear regression model. Take the data of the year 2012 as an example. Suppose that the 29 Chinese regions are arranged in conventional order, which is in fact an official order appearing in various yearbooks of China. Using the least squares calculation, we will have a linear model as below:

$$L_{i}=a+bG_{i}+\varepsilon_{i}=26.1393+0.0006388G_{i}+\varepsilon_{i}.$$

**Fig 1:**
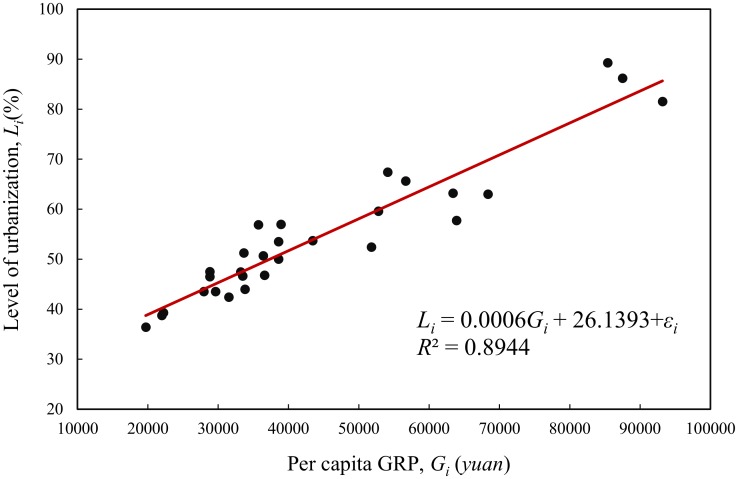
The regression model of the linear relationship between urbanization and economic development of the 29 Chinese regions (2012).

The goodness of fit is *R*^2^ = 0.8944 (Fig 1). It is easy to obtain other statistics using mathematical or statistical software.

**Fig 2:**
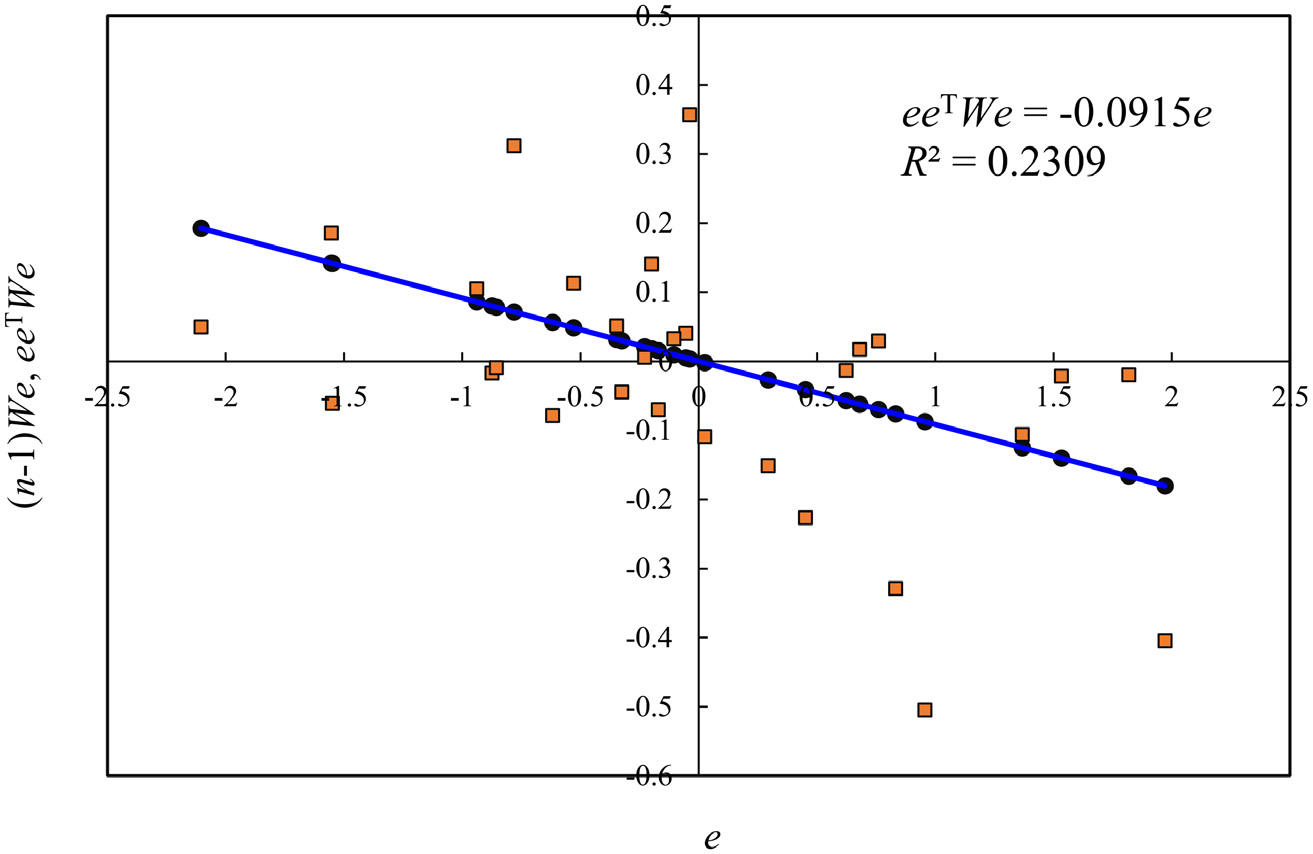
The normalized scatterplot with a trendline of serial autocorrelation for the relationship between urbanization and economic development of the 29 Chinese regions (2012).

In order to appraise the model, a test for serial correlation of residuals must be performed. Based on the standardized value of the residuals *ε*_*i*_, the Durbin-Watson statistic can be obtained using Eq 3 and the result is about *DW* = 2.2463. Then, by means of Eqs 8 and 9 and the abovementioned four-step method of calculation, we can compute SAI and RCI, and the results are *SAI* = -0.0915 and *RCI* = 2.1830. Note that the sample size *n* is substituted with degree of freedom *n*-1. The basic process and main results of calculation are attached (one calculation in S2 File). However, if we rearrange the elements of the spatial sample, the RCI value will not change, but the DW value will be different. For example, arranging the 29 regions in alphabetical order, we will have *DW* = 1.9071 and *RCI* = 2.1830. The RCI value is constant, but the DW value depends on the arrangement of regions (Table 1). The corresponding computation process and results are attached (another calculation in S3 File). Using *e* as the *x*-axis, and *ee*^T^*We* and (*n*-1)*We* as the *y*-axis, we can draw a normalized autocorrelation scatterplot of residuals as follows (Fig 2). The slope of the trendline is just equal to the SAI value, -0.0915.

The above method can be applied to the datasets of different years, from 2000 to 2012, and thus we will have 10 study cases (The statistical data of the level of urbanization from 2001 to 2004 are absent from the website of China’s NBS). The weight functions are adopted to generate spatial contiguity matrixes. One is the inverse power function, *v*_*ij*_ = 1/*r*_*ij*_, and the other is a negative exponential function in the form *v*_*ij*_ = exp(-2*r*_*ij*_/$\bar{r}$), where $\bar{r}$ denotes the average distance. This study relies heavily on the inverse power function. The calculations based on the negative exponential function are for reference only. All the results are tabulated in Table 2. The RCI values are independent of order of the 29 regions, but they are dependent to a degree on the spatial weight function. However, the Durbin-Watson statistic values depend to a great extent on the order of sample data. For example, for the year of 2000, the Durbin-Watson statistic based on conventional order of regions is *DW* = 1.5758, while the result based on alphabetical order is *DW* = 2.4939; for 2008, the two *DW* values are 1.4310 and 1.9203, respectively. The alphabetical order and conventional order are two examples. There are various other arrangements for the 29 regions. Generally speaking, for *n* geographical elements (cities or regions), we have *n*! permutations. This suggests that we can get about 29!≈8.8418*10^30^ DW values. Sometimes the differences between the numerical values of the Durbin-Watson statistic based on different permutations are considerably large. However, for a given weight function, the RCI value is uniquely determined. Changing the weight function yields different RCI values. But generally speaking, there is no significant difference between the RCI values based on different weight functions. In short, the RCI value depends to some extent on weight functions but is independent of the arrangement of elements.

**Table 2:** The Durbin-Watson statistics, RCI values, and ARCI values of residual series from linear squares regression of 29 Chinese regions (2000–2012).

Year	Arrangement in conventional order					Arrangement in alphabetical order				
		Power law based		Exponential law based			Power law based		Exponential law based	
	DW statistic	RCI	ARCI	RCI	ARCI	DW statistic	RCI	ARCI	RCI	ARCI
**2000**	1.5758	1.7576	1.7945	1.7493	1.7105	2.4939	1.7576	1.7945	1.7493	1.7105
**2005**	1.4621	1.7984	1.6745	1.8112	1.6243	1.9905	1.7984	1.6745	1.8112	1.6243
**2006**	1.5054	1.8135	1.6855	1.8352	1.6472	1.9345	1.8135	1.6855	1.8352	1.6472
**2007**	1.6049	1.8390	1.7364	1.8610	1.7029	1.9613	1.8390	1.7364	1.8610	1.7029
**2008**	1.4310	1.9045	1.7797	1.9168	1.7441	1.9203	1.9045	1.7797	1.9168	1.7441
**2009**	1.6044	1.9986	1.8986	1.9953	1.8635	1.8789	1.9986	1.8986	1.9953	1.8635
**2010**	1.8956	2.0418	1.9807	2.0240	1.9570	2.0448	2.0418	1.9807	2.0240	1.9570
**2011**	2.1046	2.1363	2.1068	2.0921	2.0565	1.9245	2.1363	2.1068	2.0921	2.0565
**2012**	2.2463	2.1830	2.1435	2.1329	2.0829	1.9071	2.1830	2.1435	2.1329	2.0829
**2013**	2.2524	2.2142	2.1755	2.1656	2.1055	1.8315	2.2142	2.1755	2.1656	2.1055

**Note**: “Power law based” means that the spatial contiguity matrix is generated with the inverse power function indicating of power-law decay. “Exponential law based” means that the contiguity matrix is generated with a negative exponential function indicating exponential decay.

### 3. Testing for serial correlation of log-linear regression analyses

It is possible that the relationship between the level of urbanization and the level of economic development is not a real linear relationship. The reason for this is that the proportion of urban population has a clear *lower limit* (0) and a strict *upper limit* (1 or 100%). If a sample size is large enough, the distribution trend of the level of urbanization dependent on per capita GRP will be a sigmoid curve instead of a straight line and can be described with a squashing function. Three equations can be employed to describe the relationship between urbanization and economic development. The first is the single logarithmic linear relation, which can be modeled with a logarithmic function [21, 22]; the second is the double logarithmic linear relation, which can be modeled with a power function [23, 24]; and the third is the logit linear relation, which can be modeled with a logistic function [25]. In fact, the urban-rural ratio of regional population conforms to the logit transform. Therefore, the relationship between the level of urbanization and per capita GRP of the 31 Chinese regions satisfies a logistic function [25], which can be expressed as

$$L_{i}=\frac{L_{\text{max}}}{1+Ae^{-kG_{i}}},$$ (27)

which can be transformed into a logarithmic linear relation, namely, ln(*L*_max_/*L*_*i*_-1) = ln*A*-*kG*_*i*_, where *L*_*i*_ and *G*_*i*_ denote the level of urbanization and per capita GRP of the *i*th regions, *A*, *k*, and *L*_max_ are parameters. Among these parameters, *L*_max_ is the capacity of the level of urbanization in a region. For simplicity, let *L*_max_ equal 100%. A least squares calculation using the 2012’s datasets consisting of 29 elements yields the following model

$$\text{ln}(\frac{100}{L_{i}}-1)=1.1201-0.00003022G_{i}+\varepsilon_{i}.$$

**Fig 3:**
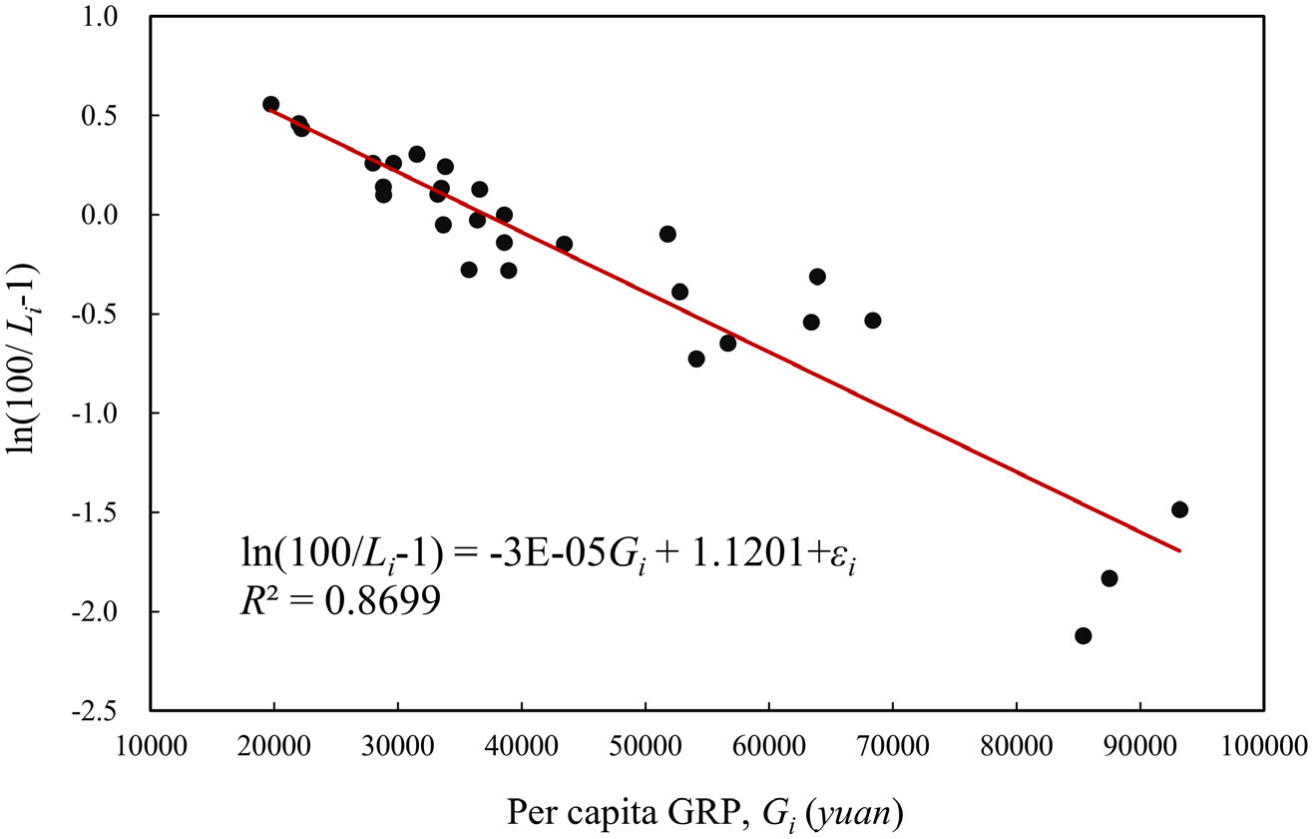
The linear regression of the logistic relationship between urbanization and economic development of the 29 Chinese regions (2012).

The goodness of fit is about *R*^2^ = 0.8699, which is less than the coefficient of determination of the linear model (Fig 3). The logarithmic linear regression can be applied to all the available datasets from 2000 to 2013 (Table 3). From 2000 to 2008, the goodness of fit of the logistic model is greater than that of the linear model, but from 2009 to 2013, the *R* square of the linear model exceeds that of the logistic model. This suggests a complicated and evolving correlation between urbanization and economic development.

**Table 3:** The coefficients and goodness of fit of the regression models of the correlation between urbanization and economic development of 29 Chinese regions (2000–2013).

Model	Parameter /Statistic	2000	2005	2006	2007	2008	2009	2010	2011	2012	2013
**Linear model**	*a*	20.1216	24.3466	25.1037	25.7844	24.6789	25.3256	25.1019	25.1020	26.1393	27.1009
	*b*	2.2724E-03	1.3107E-03	1.1510E-03	9.8978E-04	9.1474E-04	8.5882E-04	7.8448E-04	6.9522E-04	6.3884E-04	5.9096E-04
	*R*^2^	0.8358	0.8931	0.8925	0.8969	0.9068	0.9048	0.9172	0.9063	0.8944	0.8889
**Logistic model**	*k*	1.0616E-04	6.1488E-05	5.3919E-05	4.6466E-05	4.2704E-05	4.0097E-05	3.6911E-05	3.2750E-05	3.0217E-05	-2.8144E-05
	*A*	3.8269	3.1806	3.0773	2.9992	3.1543	3.0745	3.1538	3.1791	3.0651	2.9713
	*R*^2^	0.8656	0.9126	0.9109	0.9142	0.9081	0.9002	0.9057	0.8858	0.8699	0.8611

The method of spatial autocorrelation analysis can be applied to the residuals from the logistic models for different years. The results are tabulated below (Table 4). The cases are similar to those of linear models (Table 2). The Durbin-Watson values depend on the permutation of the 29 regions. For example, for 2000, the DW value based on the conventional order is about 1.5870, but the result based on the alphabetical order is around 2.4902. There is a significant difference between the two numerical values. However, without exception, the RCI value and ARCI values are free from the influence of the arrangement order of the members in the datasets. This implies that the new approach of serial correlation test applies to least squares regression based on the linearized expressions of nonlinear models.

**Table 4:** The Durbin-Watson statistics, RCI values, and ARCI values of residual series from linearized logistic models of 29 Chinese regions (2000–2013).

Year	Arrangement in conventional order					Arrangement in alphabetical order				
		Power law based		Exponential law based			Power law based		Exponential law based	
	DW statistic	RCI	ARCI	RCI	ARCI	DW statistic	RCI	ARCI	RCI	ARCI
**2000**	1.5870	1.7934	1.8068	1.7765	1.7322	2.4902	1.7934	1.8068	1.7765	1.7322
**2005**	1.3898	1.8706	1.8061	1.8782	1.7742	1.9284	1.8706	1.8061	1.8782	1.7742
**2006**	1.4574	1.8935	1.8448	1.9032	1.8215	1.8541	1.8935	1.8448	1.9032	1.8215
**2007**	1.5653	1.9246	1.9013	1.9331	1.8851	1.8928	1.9246	1.9013	1.9331	1.8851
**2008**	1.5630	2.0557	2.0749	2.0303	2.0297	1.9364	2.0557	2.0749	2.0303	2.0297
**2009**	1.7473	2.1454	2.1954	2.1034	2.1462	1.8958	2.1454	2.1954	2.1034	2.1462
**2010**	1.9178	2.1946	2.3054	2.1288	2.2451	1.9866	2.1946	2.3054	2.1288	2.2451
**2011**	2.0921	2.2599	2.3915	2.1765	2.3067	1.8872	2.2599	2.3915	2.1765	2.3067
**2012**	2.2132	2.2788	2.3946	2.1977	2.3067	1.8714	2.2788	2.3946	2.1977	2.3067
**2013**	2.2334	2.2998	2.4204	2.2219	2.3274	1.8127	2.2998	2.4204	2.2219	2.3274

## Discussion

### 1. Basic framework of methodology

**Fig 4:**
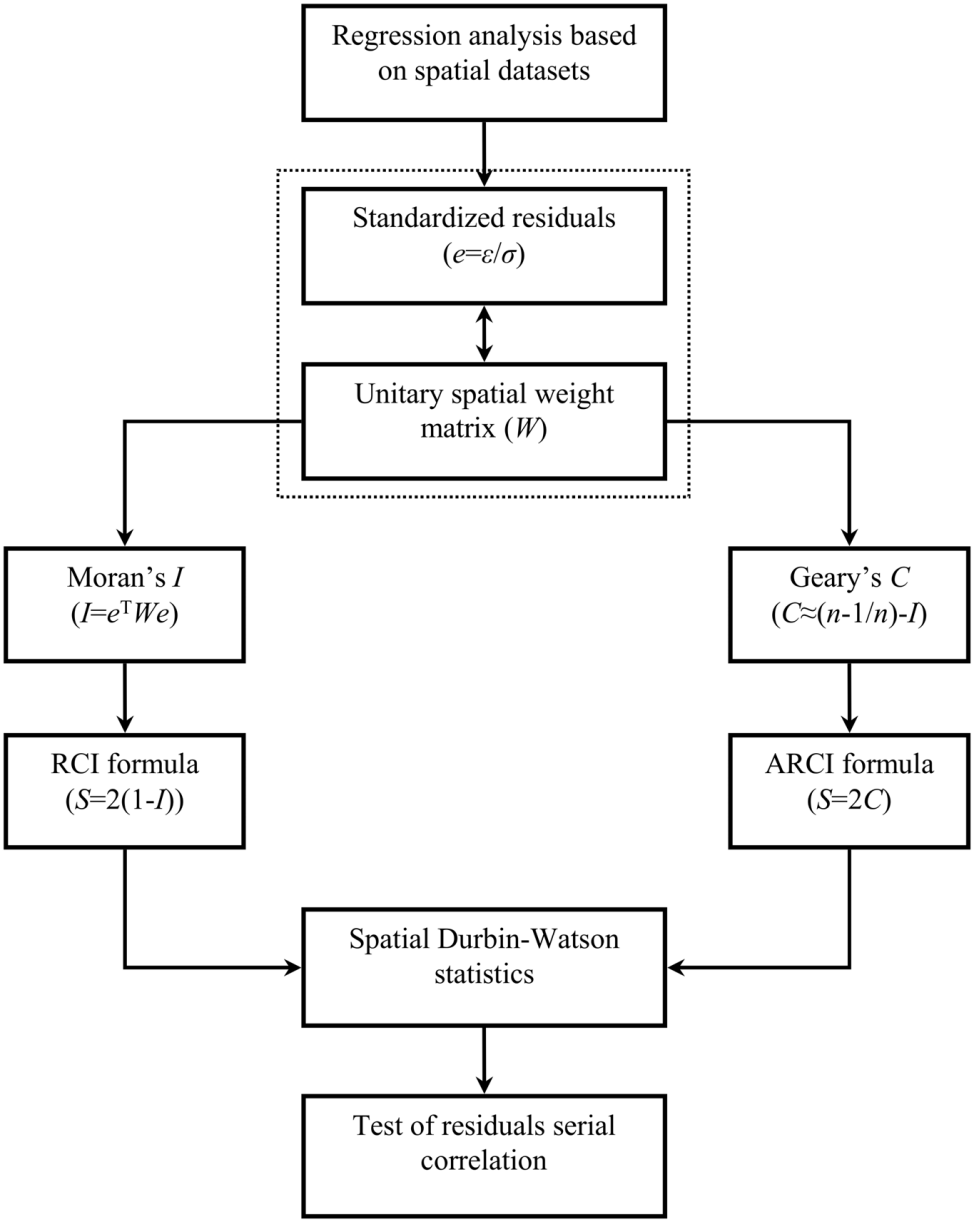
A flow chart of the two spatial autocorrelation approaches to testing residuals from least squares regression based on spatial random samples.

This paper is devoted to developing a methodology of serial correlation test for the predicted residuals from regression models based on spatial random samples. The study’s aim is method development rather than empirical analysis. Mathematical modeling is not the main task of this work, but regression analysis can be employed to show how to apply spatial autocorrelation approaches to testing for serial correlation in least squares regression. Differing from the conventional Durbin-Watson statistic, the spatial DW statistics based on Moran’s index and Geary’s coefficient, RCI and ARCI, are independent of the permutation of elements in a dataset. This indicates that the new method is effective for testing residuals from least squares regression associated with spatial modeling. The merits of this method are as follows. First, the mathematical principles are simple and easy to understand; second, the calculation is simple and convenient to implement. We can calculate RCI with MS Excel (instruction in S4 File). Of course, we can write computer programs for RCI and ARCI in Matlab (programs in S5 File). The processes of testing for serial correlation on a spatial random sample can be illustrated using a flow chart (Fig 4).

In fact, preliminary progress was made forty years ago, but the result failed to catch people’s attention. Cliff and Ord [15] once employed Moran’s index to test the regression residuals for autocorrelation [16]. However, the method was not developed further. Several advances are presented in this article. First, both Moran’s index and Geary’s coefficient are adopted to evaluate autocorrelation of regression residuals; Cliff and Ord [15] only used Moran’s index. Second, two new statistics are defined by analogy with the Durbin-Watson statistic, and the application can be associated with the ordinary Durbin-Watson test. Based on Moran’s index and Geary’s coefficient, the statistics termed RCI and ARCI for short are constructed, and, the Durbin-Watson significance tables can be utilized to make a judgment. Third, the relationships between different statistics are revealed by mathematical transformation. In this manner, it is easy to understand these statistics. Fourth, the new statistics are expressed with a matrix and a vector. The weight matrix is unitized, and the vector is standardized. So the expressions are normalized and it is convenient to compute the newly defined statistics. Fifth, typical case studies are made to demonstrate the analytic processes, and readers can follow these examples to make serial autocorrelation tests for the regression residuals based on spatial datasets.

Scientific innovation includes substantive innovation and formal innovation. The former is to discover, invent, or present new things (e.g., phenomena, relations, laws, principles, models, theories, methods, and so on), and the latter is to improve, reconstruct, develop, or simplify the existing things (models, theories, methods, etc.). The substantial innovation has been attracting people’s attention, but the formal innovation seems not to receive due attention from general researchers. However, the formal innovation is very significant in many cases. For example, the Hindu-Arabic numerals are a type of formal innovation, compared with the Roman numerals and other numerals [26]. The former makes arithmetical calculations easier. For the least squares regression, the *p*-value is a kind of formal innovation, compared with the *t*-statistic [27]. The former makes *t*-test of regression coefficients simpler [5]. For the logistic regression [28], Nagelkerk’s coefficient of strength of association is a sort of formal innovation [29], compared with Cox-Snell’s index of strength of association [30]. The former makes the association strength clearer [5]. This study presents mostly formal innovation, including both intensive innovation (e.g., reconstruction of the mathematical expressions, simplification of analytical process) and extensive innovation (e.g., construction of new indices). However, this work also possesses substantive innovation. In methodology, a set of SDW statistics are defined based on Moran’s index and Geary’s coefficient. Compared with the common Durbin-Watson statistic, the SDW statistic can be used to test spatial serial correlation of residuals; compared with the Cliff-Ord method based on Moran’s index [15], the SDW statistics are simpler, clearer, and more convenient to apply. In technique, a complete computer program based on Matlab has been written and is attached as supporting materials. Using this Matlab program, students can readily calculate RCI and ARCI.

### 2. Deficiency in the method

Any measure has its shortcomings, and any method has its flaws. The incompleteness of the SDW statistics and the corresponding test method rests with SCM. First, the RCI values and ARCI values depend on the form of the spatial weight function. Different spatial weight functions yield different SCMs, and different SCMs result in different SDW values. In geographical analysis, we have four types of spatial weight functions, including inverse power function, negative exponential function, staircase function, and semi-staircase function [17, 19]. The inverse power function is for the spatial processes based on globality, i.e., the whole of a geographical system, associated with action at a distance; the negative exponential function is for those based on localization or quasi-globality; the staircase function is for those based on locality, i.e., the parts a geographical system; and the semi-staircase function is for those based on quasi-locality [19]. In many cases, it is difficult to select a weight function. In order to choose a proper weight matrix, it is necessary to know the mathematical properties and physical meanings of different functions and the geographical features of study areas. Second, the RCI values and ARCI values rely on the definition of spatial contiguity. Different contiguity definitions yield different SWMs, and different SWMs lead to different SDW values. The spatial contiguity can be measured by spatial relationships and distances, and the spatial relationships and distances can be considered from different points of view. There exist corresponding relationships between spatial weight functions and the definitions of contiguity, which are displayed in Table 5. The two kinds of problems above-mentioned cannot be solved at present and require much more study before the effective solutions are finally found for spatial autocorrelation analysis.

**Table 5:** The relationships between spatial contiguity functions and the definitions of contiguity.

Spatial weight function	Mathematical expression	Spatial measurement	Geographical meaning
**Inverse power function**	$v_{ij}=\frac{1}{r_{ij}^{b}}$	Spatial distances	Action at a distance
**Negative exponential function**	$v_{ij}=\text{exp}(-\frac{2r_{ij}}{\bar{r}})$	Spatial distances	Semi-locality or quasi-action at a distance
**Semi-staircase function**	$v_{ij}=\left\{ \begin{array}{l} 1,\;r_{ij}\leq \bar{r}\\ 0,\;r_{ij}>\bar{r} \end{array}\right.$	Spatial distances and relationships	Semi-locality
**Staircase function**	$v_{ij}=\left\{ \begin{array}{l} 1,\text{ if }i\text{ borders }j\\ 0,\text{ others} \end{array}\right.$	Spatial relationships	Locality

The main limitation of this study rests with data quality. The numerical materials are statistical data rather than so-called big data. The quality of statistical data cannot be guaranteed in many cases because a sampling is a process of selection in a top-down way. On the contrary, big data are collected through a bottom-up approach. What is more, the analytical results of this work are not efficiently represented and displayed by spatial technology such as geographical information system (GIS). A shortage of a research is just the future directions of improvement. The SDW test can be applied to the spatial analysis based on GIS and big data.

## Conclusions

A well-known issue in spatial analysis is testing for serial correlation in least squares regression based on spatial random samples in a simple way. The aim of this paper is to solve the following problems: the conventional Durbin-Watson statistic test is simple, but it cannot be effectively applied to the random spatial serial correlation. Moran’s index or Geary’s coefficient can be applied to testing spatial serial correlation, but it is not easy to popularize spatial autocorrelation to general students, like the autocorrelation coefficient of the time series analysis cannot be popularized among common researchers. In this work, a simple methodology for testing autocorrelation of residuals is illustrated, including mathematical models, statistical principles, calculation processes, and sample cases. In addition, a complete Matlab program is attached for application and practice. The main conclusions follow.

**First, spatial autocorrelation analysis can be simplified to test the serial correlation of residuals from least squares regression.** The formula of the Durbin-Watson statistic is a mathematical expression based either on one-order time lag for time series or on one-step spatial displacement for ordered space series. If we make a regression analysis using cross-sectional data from spatial random sampling, the Durbin-Watson test will be ineffective because the results depend on the arrangement order of elements in arrays. Rearranging the data sequences in the independent variable(s) and dependent variables will yield a range of DW values. In many cases, these DW values are significantly different from one another. If we use the spatial weight function to replace the parameter of time lag or space displacement, the problem of random results will be well solved. Based on Moran’s index and Geary’s coefficient, a set of spatial Durbin-Watson statistics can be defined to test for serial correlation of random spatial residuals.

**Second, the new statistics for testing residual correlation of spatial random series can be constructed in two related ways.** One is by analogy with Moran’s index, and the other is by analogy with Geary’s coefficient. By way of Moran’s index, we can get a spatial autocorrelation coefficient of spatial residuals. One minus the autocorrelation coefficient (SAI) is equal to half of the Durbin-Watson statistic of residual series (RCI) in a spatial sense. In other words, doubling the difference between 1 and the SAI yields the precise RCI value. By way of Geary’s coefficient, we can obtain another spatial correlation index (SAI). Doubling this index gives an approximate RCI (ARCI), which really corresponds to the Durban-Watson statistic, and can be called SDW statistic in spatial analysis. The RCI can be applied to small spatial samples, while the ARCI is suitable for large spatial samples.

**Third, the common Durbin-Watson significance tables can be adapted for testing spatial serial autocorrelation.** The common Durbin-Watson statistic is based on a time-lag parameter, while the spatial Durbin-Watson measurements are based on a weight matrix. Using the spatial weight function to replace the time-lag parameter, and using the weighted average to replace the arithmetic mean, we derive a set of new statistics that can test for serial correlation of predicted residuals of least squares regression models. Compared with the common Durbin-Watson statistic, the SDW statistics do not differ mathematically. Thus, the Durbin-Watson significance tables in common use can be employed to determine the upper and lower bounds for the critical values of SDW statistics and make confidence statements based on certain significance levels (*α*) and degrees of freedom (*df* = *n*-*m*-1).

## Supporting Information

S1 File Datasets of per capita GRP, level of urbanization, and railway distances. This file contains the original data used in this paper.
(XLSX)

S2 File Basic processes of computing RCI for 2012 (conventional order). It provides a complete process of computing the residual correlation index (RCI) based on the conventional order of element arrangement.
(XLSX)

S3 File Basic processes of computing RCI for 2012 (alphabetical order). It provides a complete process of computing the residual correlation index (RCI) based on the alphabetical order of element arrangement.
(XLSX)

S4 File A simple approach to calculating RCI and ARCI using MS Excel. It illustrates how to calculate the residual correlation index (RCI) and approximate residual correlation index (ARCI) step by step using MS Excel.
(DOCX)

S5 File Two programs of RCI calculation based on the datasets of 2012. It provides two Matlab programs for calculating the residual correlation index (RCI): one is based on the power-law decay function, and the other is based on the exponential-decay function.
(M) Click here for additional data file.
