# Supplementary material for: Spatial Autocorrelation Approaches to Testing Residuals from Least Squares Regression
Source: PLoS One. 2016 Jan 22;11(1):e0146865. doi: 10.1371/journal.pone.0146865 (PMC4723244; doi:10.1371/journal.pone.0146865)
Supplement: S4 File — It illustrates how to calculate the residual correlation index (RCI) and approximate residual correlation index (ARCI) step by step using MS Excel. (DOCX) [file pone.0146865.s004.docx]

## A simple approach to calculating RCI with MS Excel

The residual correlation index (RCI) of the linear regression models based on spatial datasets can be calculated using MS Excel. The basic steps are similar to those that are employed to compute Moran’s index (Chen, 2013). Taking the data of 2012 of 29 Chinese regions, including provinces, autonomous regions, and municipalities directly under the central government, as an example, I will show how to compute the RCI values using what is called *three-step method*.

### 1 Regression analysis

First of all, we should make a linear regression analysis and obtain the residual series. The independent variable is the per capita *gross regional product* (GRP), and the dependent variable is the *level of urbanization* (Figure S1a). From the Summary Output, we can find the Residual Output (Figure S1b).


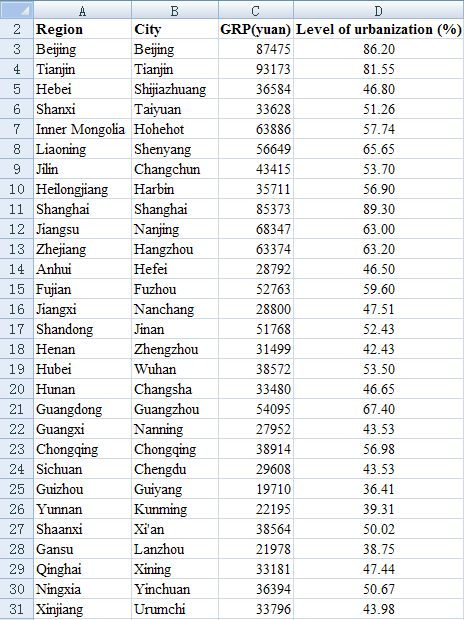

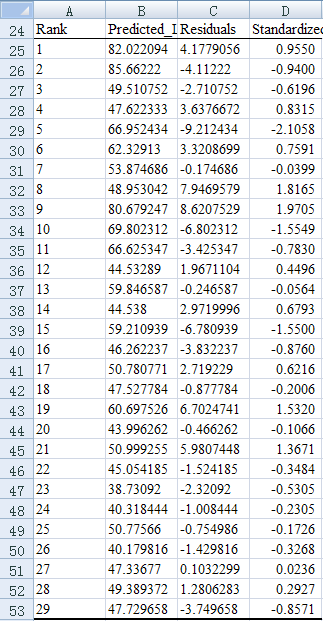


a. Observational data b. Residual series

**Figure S1** The statistical data of GRP and urbanization level of 29 Chinese regions and the residual series of linear regression analysis (2012)

### 2 Procedure of calculations based on power function

#### 2.1 Step 1: standardizing size measures

For the residual autocorrelation analysis of linear regression models, this step can be omitted because the Residual Output of MS Excel includes standardized residuals (Figure S1b). These standardized residuals are based on *sample standard deviation* (SSD) rather than *population standard deviation* (PSD).

For the further calculations, the railway distance matrix of Chinese capital cities is prepared as below (Figure S2).


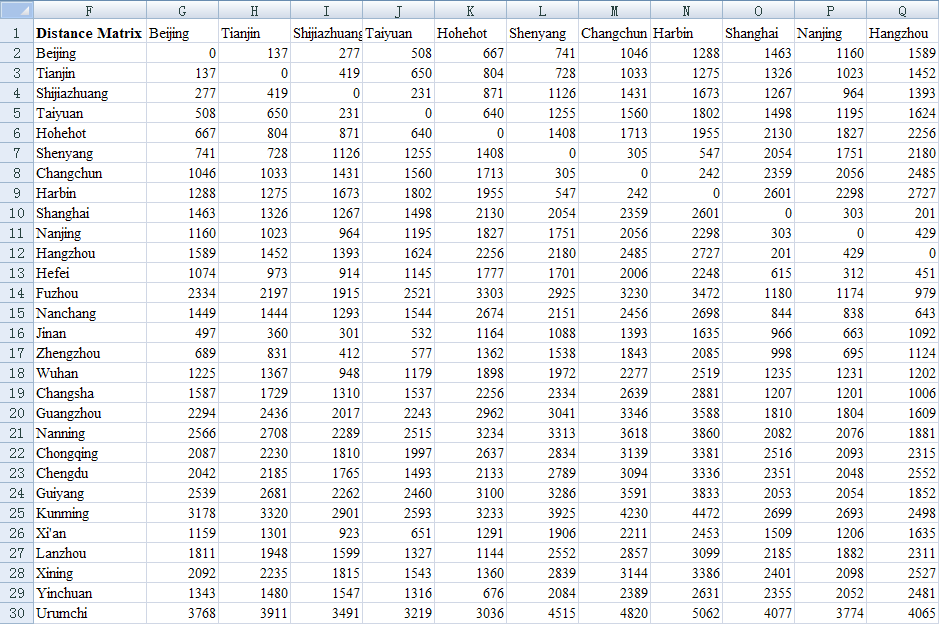


**Figure S2** The distance matrix of 29 Chinese cities by railway network (partial results)

#### 2.2 Step 2: generating normalized spatial weights matrix

The process of generating a spatial weights matrix comprises three steps as follows.

**(1) Convert the spatial distance matrix into a spatial contiguity matrix.** Select a distance-decay function based on inverse power law such as

$v_{ij}=\left\{\begin{matrix} r_{ij}^{-1},\text{ }i\neq j \\ 0,\text{ }i=j \end{matrix}\right.$, (1)

where *r_ij_* denotes the distance between city *i* and city *j*, and *v_ij_* represents the spatial contiguity measurement. The railway distance matrix is shown in Figure S2. In cell G33, input a formula such as “=IF(G2=0,0,1/G2)”, press **Enter**, it will yield a number 0. Seizing the lower right corner of cell G33 with the mouse, and dragging it right and down, we can generate all the spatial contiguity values, which are partially shown in Figure S3.


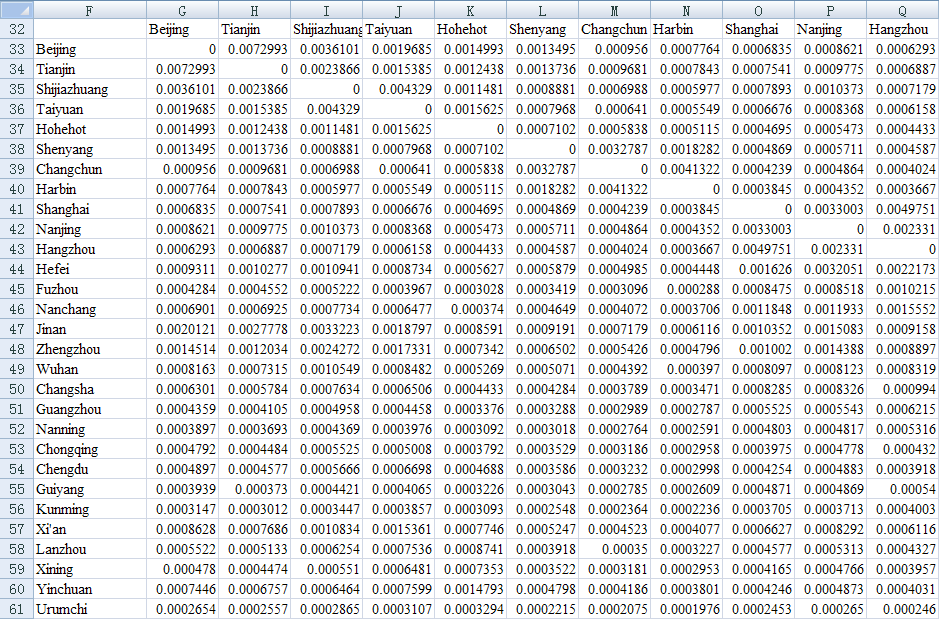


**Figure S3** Spatial contiguity matrix of 29 Chinese capital cities based on power function (partial results)


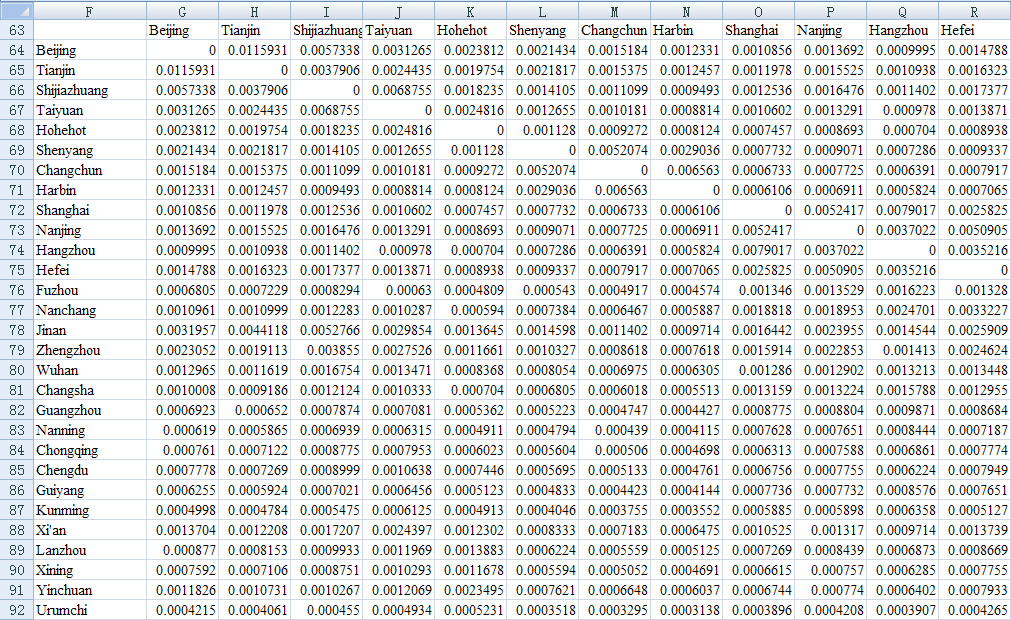


**Figure S4** Normalized spatial weights matrix of 29 Chinese capital cities based on power function (partial results)

**(2) Summate the spatial contiguity values.** The formula is as below:

$S=\sum_{i} \sum_{j} v_{ij}$, (2)

where *S* denotes an amount obtained by double summation. In cell AI62, input a formula “=SUM(G33:AI61)”, press **Enter**, yield a sum about 0.6296.

**(3) Transform the spatial contiguity matrix into a *normalized spatial weights matrix* (*W*).** In cell G64, input a formula “=G33/$AI$62”, press **Enter**, and it will yield the first value of spatial weights. Catching the bottom right corner of cell G64, pulling it right and down, we can produce all the values of the normalized spatial weights matrix, which are displayed in Figure S4 (see the file S2 for details).

#### 2.3 Step 3: computing RCI

By means of the formula *I*=*e*^T^*We*, we can calculate Moran’s index (*I*_s_) based on SSD using Excel functions “mmult” and “transpose” easily. Suppose the standardized residuals are distributed from D2 to D30, and the normalized weight matrix is distributed from G64 to AI92. According to the number and result arrangement in the same worksheet, in any cell, say, C34, we can input a formula as follows:

“=MMULT(MMULT(TRANSPOSE(D2:D30),G64:AI92),D2:D30)”

Pressing three keys **Ctrl**, **Shift** and **Enter** at the same time yields the SSD-based Moran’s index immediately. The value is about *I*_s_ =-0.0915. Thus, the RCI can be computed using the formula *S*=2(1-*I*_s_), and the result is *S*=2*(1+0.0915)= 2.1830 (see the file S2 for details).


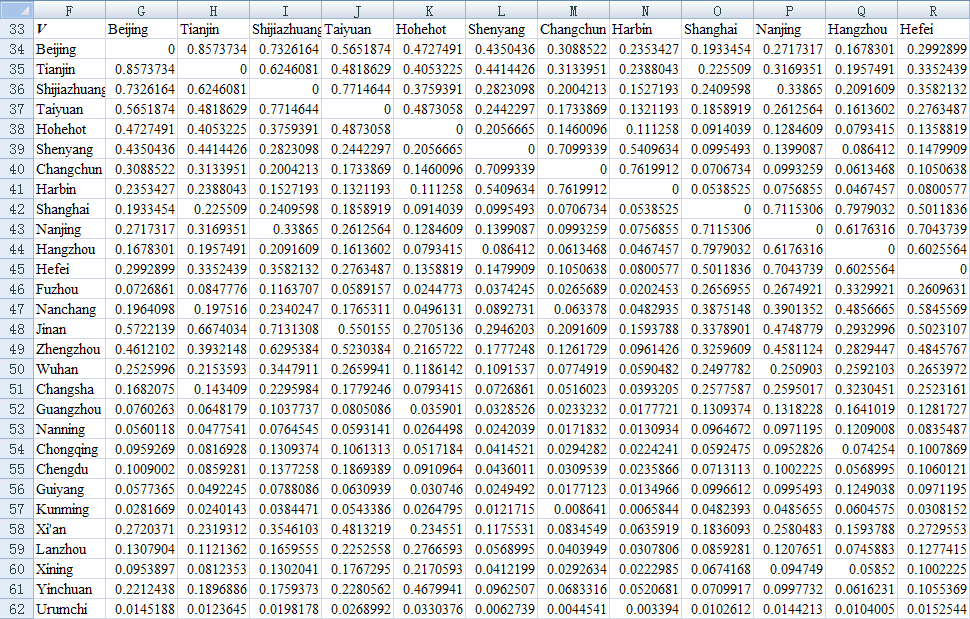


**Figure S5** Spatial contiguity matrix of 29 Chinese capital cities based on exponential function (partial results)

### 3 Procedure of calculations based on exponential function

We have more than one way to generate a spatial contiguity matrix (Chen, 2012). In step 2 of Section 2, the power function can be replaced with an exponential function to define a distance-decay function. The standard form of a negative exponential function is as below:

, (3)

where denotes the average distance, or the mean value of the elements in the distance matrix. In cell G34, input a formula “=IF(G3=0,0,EXP(-2*G3/$AI$32))”, press **Enter**, it will yield a number 0. Seizing the bottom right corner of cell G34 and dragging it right and down produce all the spatial contiguity values based on the negative exponential function, which are shown in Figure S5. Then, summate the spatial contiguity values using equation (3). In cell AI63, input a formula “=SUM(G34:AI62)”, press **Enter**, yield a sum about 154.04184. Finally, transform the spatial contiguity matrix into a normalized spatial weights matrix, which are shown in Figure S6. The other and rest steps are the same as those illustrated in Section 2. The SSD-based Moran’s index is about *I*_s_ =-0.0664, and the RCI is *S*=2*(1+0.0664)= 2.1329 (see the file S2 for details).


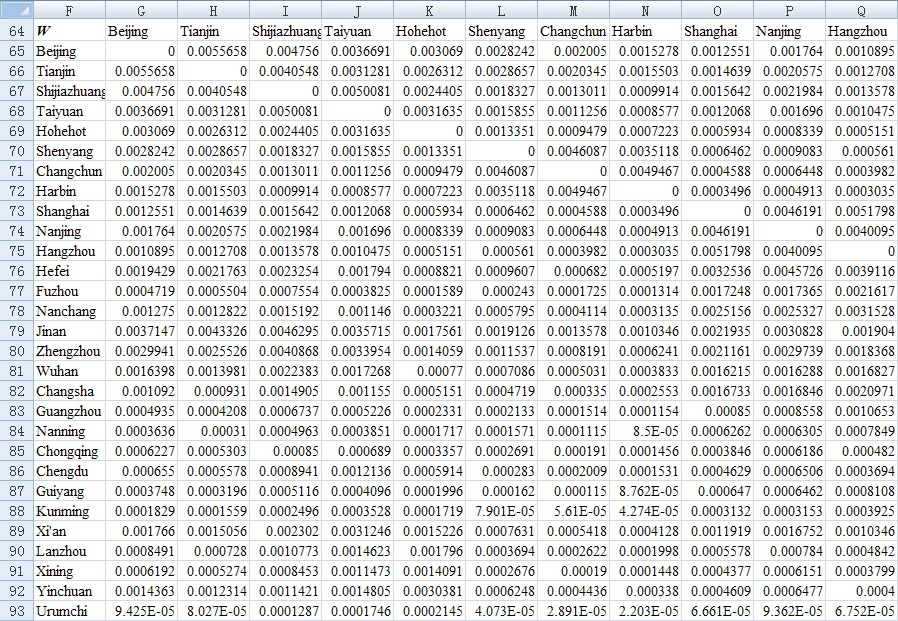


**Figure S6** Unitized spatial weights matrix of 29 Chinese capital cities based on exponential function (partial results)

### References

Chen YG (2012). On the four types of weight functions for spatial contiguity matrix. *Letters in Spatial and Resource Sciences*, 5(2): 65-72

Chen YG (2013). New approaches for calculating Moran’s index of spatial autocorrelation. *PLoS ONE*, 8(7): e68336
